# Ileal perforation involvement in Wegener granulomatosis comorbid with COVID-19 infection: A case report and review of the literature

**DOI:** 10.1097/MD.0000000000036973

**Published:** 2024-01-26

**Authors:** Huijuan Shao, Dong Liu, Xiaofeng Zheng, Jiucong Zhang, Wenbo Li, Peng Chen, Zhen Qian, Jie Yang, Dongmei Liu

**Affiliations:** a Department of Gastroenterology, The 940 Hospital of Joint Logistic Support Force of PLA, Lanzhou, China; b Department of Intensive Care Unit, The 940 Hospital of Joint Logistic Support Force of PLA, Lanzhou, China; c Department of Gastroenterology, Second Clinical Medical College of Lanzhou University, Lanzhou, China; d Department of General Surgery, The 940 Hospital of Joint Logistic Support Force of PLA, Lanzhou, China; e Department of Pathology, The 940 Hospital of Joint Logistic Support Force of PLA, Lanzhou, China.

**Keywords:** antineutrophil cytoplasmic antibodies, case report, gastrointestinal involvement, granulomatosis with polyangiitis (GPA), ileal perforation, literature review

## Abstract

**Rationale:**

Granulomatosis with polyangiitis (GPA) is a systematic autoimmune disease. The typical clinical involvement of GPA entails the upper and lower respiratory tracts, and the kidneys. Gastrointestinal (GI) involvement is uncommon and unless detected and treated promptly, may lead to life-threatening complications such as perforation. We aim to review all available publications since the first description in 1982 dealing with GI perforation in patients with Wegener granulomatosis and draw attention to this serious situation.

**Patient concerns:**

We present a 54-year-old man diagnosed with GPA who presented initially with nasal symptoms and suffered ileal perforation following Corona Virus Disease 2019 infection. We also review previously reported patients with Wegener granulomatosis who had GI perforation to investigate the perforation site and period, pathology, diagnosis, and treatment methods.

**Diagnoses and Interventions:**

The case of a GPA-diagnosed patient who presented initially with nasal symptoms and suffered ileal perforation following Corona Virus Disease 2019 infection. We recommended a renal puncture biopsy, steroids, and immunosuppressants to improve the patient condition. The patient and his family refused these treatment recommendations.

**Outcomes:**

Our patient exhibited continued progressive vascular inflammatory changes and eventual irreversible systemic damage. These sequelae were attributed to the patient declining prednisolone and immunosuppressant therapy.

**Lessons:**

GI perforation is rare in GPA but severe complication. Consequently, we recommend that early diagnosis and treatment with steroid hormones and immunosuppressants for GPA patients with GI perforation.

## 1. Introduction

Granulomatosis with polyangiitis (GPA), also known as Wegener granulomatosis, is a systematic autoimmune disease with multisystem involvement. GPA has an unknown etiology but is classified pathologically as an antineutrophil cytoplasmic antibody (ANCA)-associated vasculitis. It is characterized histopathologically by the presence of granulomas, necrosis, and vasculitis.^[1]^ GPA involves small-medium blood vessels. Typically, affected vessels are located predominantly in the upper and lower respiratory tracts, and the kidneys.^[2]^ Gastrointestinal (GI) involvement is rare, especially involvement complicated with GI tract perforation. Patients with GI-involved GPA who do not receive prompt and sustained treatment have an elevated incidence of complications and a heightened risk of mortality.^[2]^

Here, we report the case of a GPA-diagnosed patient who presented initially with nasal symptoms and suffered ileal perforation following Corona Virus Disease 2019 (COVID-19) infection. The patient exhibited continued progressive vascular inflammatory changes and eventual irreversible systemic damage. These sequelae were attributed to the patient having declined prednisolone and immunosuppressant therapy. We review the literature relevant to GPA complicated with intestinal perforation to raise clinicians’ awareness of the disease and prompt appropriate treatment administration to patients that can prevent irreversible local or systemic damage.

## 2. Case presentation

**Figure 1.**
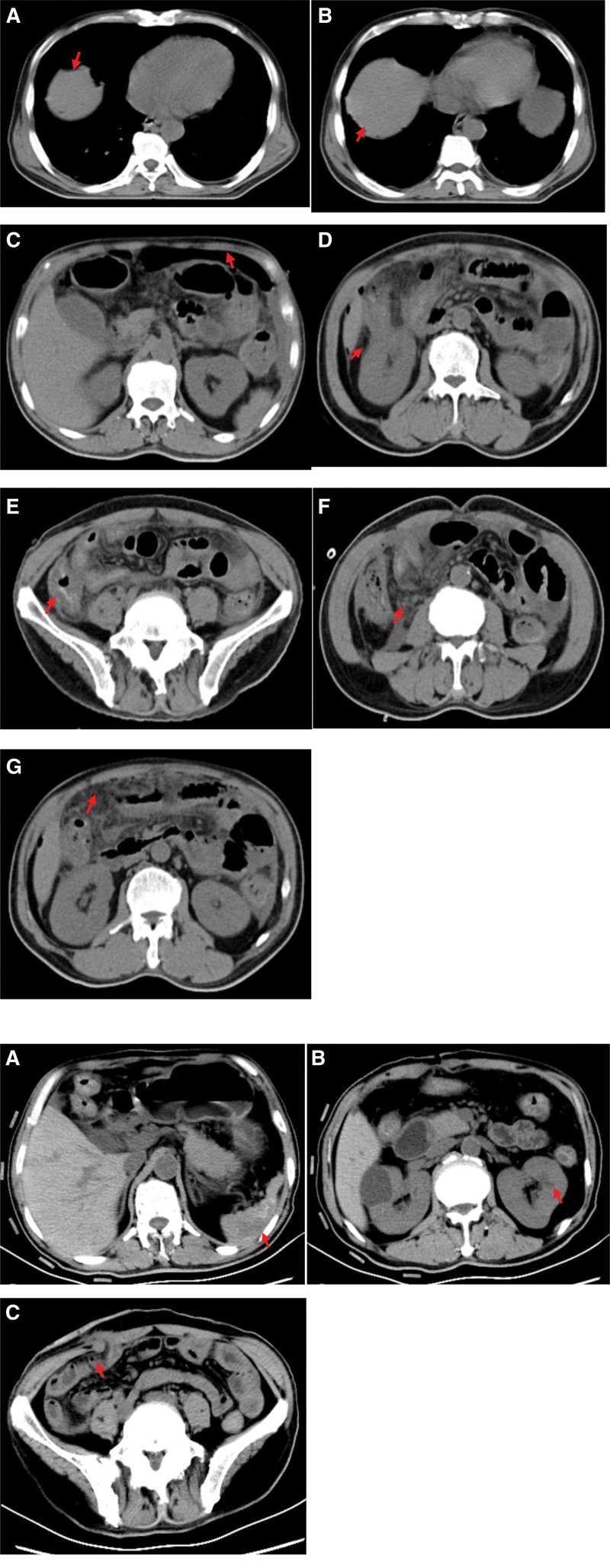
**A Preoperative computed tomography of the abdomen**. Multiple free air in subdiaphragm and abdominal cavity (A–D); edema of the intestinal wall of the ileocecal and ascending colon, with peripheral exudate and effusion in the colonic sulcus (E and F); peritonitis (G). (B) **Postoperative computed tomography of the abdomen**. Lamellar hypodense shadow in the spleen (A); patchy hypointense shadow in the renal parenchyma (B); high-density shadow in the intestinal wall at the stump of the hepatic flexure of the colon, with a little exudate around it; the stoma was unobstructed after a right lower abdominal fistula (C).

**Figure 2.**
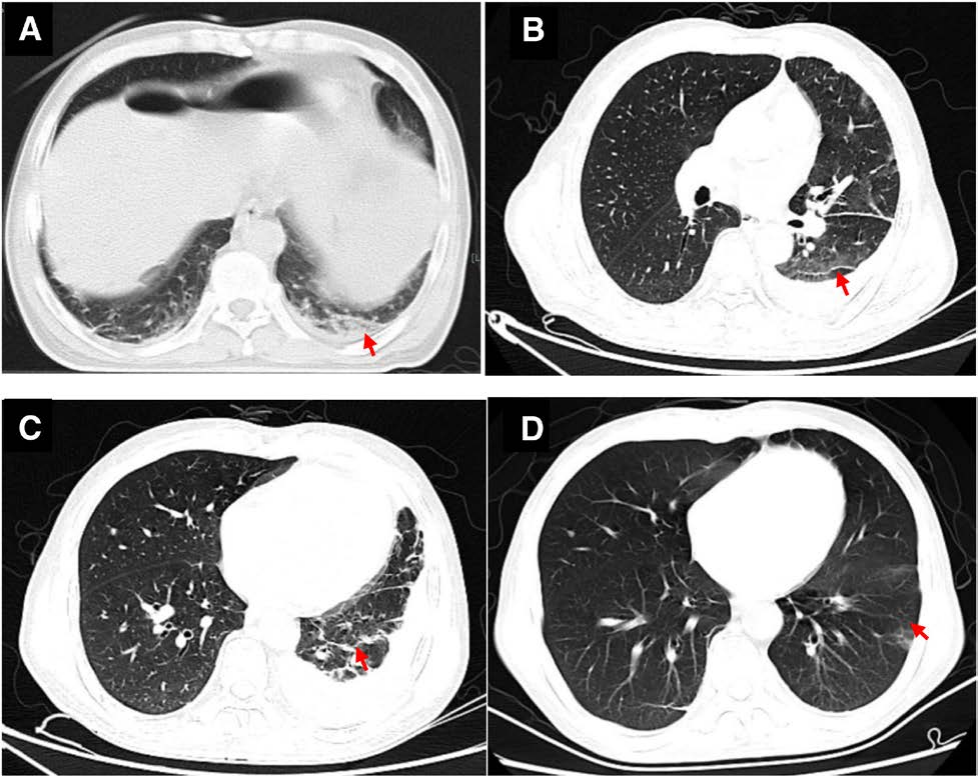
**Computed tomography of lungs**. Inflammatory exudate in both lower lobes of the lungs (A, preoperative);Multiple flaky and patchy shadows in the apical right lung, lower lobe of both lungs and upper lobe of the left lung (B and C, preoperatively); patchy ground-glass opacity under the left lung subpleural (D, postoperative).

**Figure 3.**
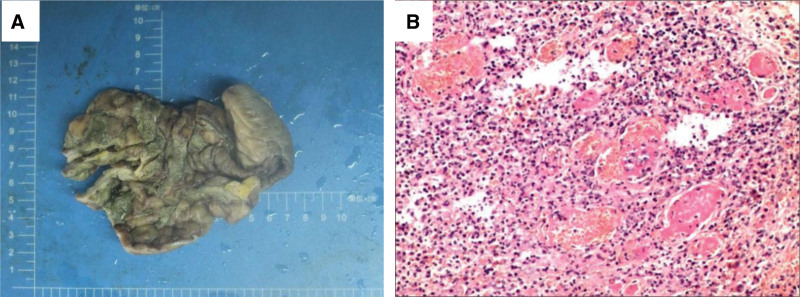
**Ileocecal canal and part of ileum.** Erosion and necrosis of the colonic mucosa, with most of it disappearing and showing geographic changes (A); deformation, erosion, and necrosis of some areas of intestinal wall tissue and mucosa, disruption of intestinal wall continuity in focal areas, and infiltration of epithelioid and inflammatory cells (B); consistent with an ulcer with intestinal perforation surrounded by purulent inflammatory changes (HE, ×200).

**Figure 4.**
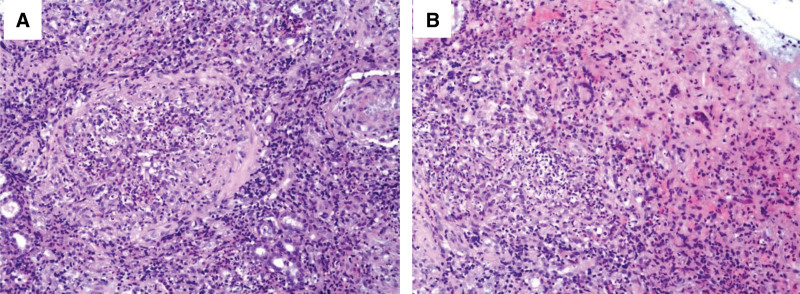
**The biopsy of nasal mucosal tissue.** Massive necrotic material and inflammatory granulation tissue, interstitial infiltration of numerous inflammatory cells, localized necrotizing vasculitis changes (A), epithelioid cells and multinucleated giant cells are seen (B); **immunohistochemistry**: CD3 (T lymphocyte+), CD5 (−), CD20 (B lymphocyte+), CD34 (vascular+), SMA (vascular+), Desmin (smooth muscle+), CD68 (histiocyte+), Ki67 (hot spot area index 30%), CD56 (−), granzyme B (scattered+), CD2 (T lymphocyte+) S100 (−), Langerin (−). **Special staining results**: PAS (−), antacid (−). **Molecular pathology results**: EBER (−) (HE, ×200).

a 54-year-old man was admitted to our hospital on December 24, 2022 with right lower abdominal pain that had commenced 15 hours prior. Anamnesis indicated that the patient had nasal swelling that had persisted for ≥ 3 months and bilateral lower limb edema that had persisted for 2 months. The patient blood pressure was 110/70 mm Hg and he had full abdominal pressure, abdominal muscle tension, and diminished bowel sounds (about 1 sound per minute). An emergency abdominal computed tomography (CT) was suggestive of free gas in the abdomen, a finding consistent with a GI tract perforation (Fig. 1A). A chest CT showed strip shadows in the lower lobes of both lungs, suggesting atelectasis combined with inflammation (Fig. 2A). The patient serum creatinine concentration was 938 μmol/L and he tested positive for SARS-CoV-2 nucleic acids. He was sent via green channel routing to the emergency department operating room for an exploratory laparotomy. Intraoperatively, the patient was found to have a perforation 5 cm above the ileocecal region, with fecal leakage from the rupture and severe edema of the surrounding intestinal wall. Perform ileocecal resection, ileostomy, and abdominal drainage on the patient. The pathology of the resected ileocecal intestine showed inflammatory vascular changes (Fig. 3). Postoperative CT of the abdomen showed low-density shadows in the spleen and kidney, suggesting vasculitis and local infarction. The stoma was unobstructed after a right lower abdominal fistula was performed (Fig. 1B). The patient had been admitted previously to our nephrology department on October 9, 2022, at which time he had tested positive for cytoplasmic type cytoplasmic antineutrophil cytoplasmic antibody (c-ANCA) and anti-proteinase 3 antibody type antineutrophil cytoplasmic antibodies proteinase 3 (PR3-ANCA). His chest CT showed multiple solitary and patchy shadows in the apical right lung, lower lobes of both lungs and upper lobe of the left lung, suggesting lung infection (Fig. 2B and C). Immunohistochemical analysis of a nasal mucosa tissue biopsy together with the patient clinical history was suggestive of GPA (Fig. 4). The patient laboratory data from his admission to discharging are summarized in Table 1.

**Table 1:** Patients’ laboratory data.

Date	WBC (×10^9^/L)	NEU (×10^9^/L)	LYM (×10^9^/L)	HGB (g/L)	PCT (ng/mL)	IL-6 (pg/mL)	APTT (s)	d-Dimer (mg/L)	CRE (umol/L)	ALB (g/L)
2022.12.24	17.03	16.43	0.38	75			33.60		938	23.20
2022.12.25	16.20	15.86	0.19	62	>100.00	1021.00	35.30	8.64	858	19.20
2022.12.26	16.54	15.73	0.41	67					242	
2022.12.27	15.33	14.17	0.62	65	68.99	27.60			177	
2022.12.28	12.81	11.74	0.55	66		36.64	23.90			
2022.12.29	12.08	10.52	0.93	66	15.56	51.40	30.50	9.83	207	28.80
2022.12.30	13.67	11.84	1.23	69	5.61	32.20	32.20	13.57	132	30.20
2023.1.01	11.40	8.85	1.61	66					318	33.50
2023.1.03	18.28	15.70	1.44	69	1.11	147.90				
2023.1.04	19.88	17.49	1.22	62					400	32.10
2023.1.06	12.57	10.22	1.31	48						
2023.1.08	11.41	9.53	1.15	47					360	27.20
2023.1.10	14.85	11.41	2.67	19					390	23.10

Normal value reference range: WBC: (3.97–9.15) × 10^9^/L, NEU: (2.00–7.00) × 10^9^/L, LYM: (0.80–4.00) × 10^9^/L, HGB: (131–172) g/L, PCT: (0.000–0.046) ng/mL, IL-6: (0–7) pg/mL, APTT: (23.3–32.5) s, PT: (10.0–15.0) s, PT%: (70.0–130.0)%, d-dimer: (0–0.55) mg/L, CRE: (35.0–97.0) umol/L, ALB: (35.0–53.0) g/L.

The patient main postoperative diagnoses were: septic shock with multiple organ failure; ascending colon perforation with diffuse peritonitis; GPA; SARS-CoV-2 infection. The patient developed septic shock with multiple organ failures and was resuscitated in the intensive care unit. On the 5th day after the operation, his condition stabilized and he was transferred from the intensive care unit back to the general ward. We recommended a renal puncture biopsy, steroids, and immunosuppressants to improve the patient condition. The patient and his family refused these treatment recommendations. On the 15th day after the operation, dark red blood flowed from his ileostomy and he suffered hemorrhagic shock during which his hemoglobin level dropped to 47 g/L. The patient family refused further treatment again and he was discharged from the hospital. Our attempts to follow up with the patient after discharge revealed that he died 3 days after being discharged.

The study was reviewed and approved by the Institutional Review Board of The 940 Hospital of Joint Logistic Support Force of PLA according to the standards of the Declaration of Helsinki.

## 3. Discussion

ANCA-associated systematic vasculitis is a rare autoimmune disease that may present with GPA, microscopic polyarteritis, and eosinophilic granulomatosis with polyangiitis.^[3]^ The incidence of GPA is approximately 0.4 to 11.9 cases per 1 million person-years, with similar incidence rates for men and women; typically, GPA occurs in people who are 45 to 65 years old.^[4]^ The etiology and pathogenesis of GPA are not fully understood, but it is thought that infectious, environmental, chemical, toxic, and pharmacologic factors may trigger GPA pathogenesis in genetically susceptible individuals.^[5]^ Although the genetic basis of GPA has yet to be clarified, the abnormal expression of 3 human leukocyte antigen genes, namely *HLA-DPB1, PRTN3*, and *SERPINA1*, have been implicated.^[6,7]^

GPA is a rare multisystem ANCA-associated vasculitis characterized by granulomatous inflammation, pauci-immune necrotizing glomerulonephritis, and vasculitis of predominantly small vessels.^[8]^ The upper respiratory tract is involved in more than 90% of GPA cases, with the nasal cavity and paranasal sinuses being the most common sites of involvement in the head and neck.^[9]^ Clinical or histological evidence of kidney involvement has been detected in some 80% of patients with GPA.^[10]^ In a study of 18 patients who were diagnosed with GPA with nasal symptoms as the first presenting symptom, 13 were confirmed to have ANCA, including 2 patients who died from disease progression.^[11]^ Although extremely rare, GI perforation is a serious and life-threatening complication of GPA. Incidences of GI perforation in patients with GPA are noted mostly in the small intestine, where they have been associated with a high mortality rate (46.7%).^[2]^ Eriksson et al^[12]^ observed GI involvement in only 15/216 patients (6%) with GPA/microscopic polyarteritis; among them, there were 5 patients (2% of the study cohort) who had an intestinal perforation, with abdominal pain and GI bleeding being their most common GI symptoms.^[12]^

The initial symptom reported by the patient in the present case was nasal swelling, and subsequent medical investigation revealed c-ANCA presence and the apparent involvement of the lungs and kidneys. A nasal mucosal tissue biopsy confirmed a diagnosis of GPA. Two months after being admitted with GPA comorbid with COVID-19, the patient suffered an ileal perforation that caused serious complications, including diffuse peritonitis, septic shock, and multi-organ failure, and he was stabilized with emergency surgery. During our patient intestinal repair operation, vascular inflammatory changes of the viscera and localized infarction of the spleen and kidneys, consistent with the aforementioned abdominal CT signs, were evident. Inflammatory infiltration was confirmed by postoperative biopsied tissue pathology. Similarly, Akbulut reported the occurrence of splenic infarction in a patient diagnosed with GPA and hypothesized that the infarction was likely due to ischemic necrosis consequent to vasculitis lesions.^[13]^

We reviewed the English literature cataloged in the PubMed and Google Scholar databases from 1982 to 2023 and found 25 reported cases of GPA-related intestinal perforation. The clinical characteristics of those 25 patients and of the patient in the present case are summarized in Table 2.^[2,13–35]^ These patients had a wide-ranging age-of-onset (19–69 years) with a predominance of young adult patients and a male-to-female ratio of 18:8. This sample of 26 patients with GPA includes 9 patients who were PR3-ANCA–positive, 10 patients who were c-ANCA–positive, 2 patients who were positive for both PR3-ANCA and c-ANCA and a single patient who had the anti-myeloperoxidase antibody. In most of these cases (20/26; 76.92%), perforations were located along the small intestine, including 11 cases of ileal perforations only, 4 cases of perforation of both the ileum and colon, and 6 cases of colorectal perforations. Most of the patients in the presently described sample of 26 patients were found to have pulmonary lesions (N = 22), with lesions of the ear, nose, and/or throat (N = 16) and renal lesions (N = 14) also being observed fairly commonly. In a factor analysis aimed at identifying possible survival-related factors among these 26 patients with GPA complicated with GI perforation (Table 3), we found that survivors and deceased patients did not differ significantly with respect to sex, age, ANCA positivity, lung and kidney lesion incidence, or time of bowel perforation onset. The proportion of the survival group with ear, nose, and throat involvement was significantly higher than that for the deceased group (*P* = .046). It is possible that the presence of lesions of the ear, nose, and throat, unlike the involvement of lung and kidney lesions, enabled the disease to be detected at an early stage, allowing for more timely diagnosis and treatment.

**Table 2:** Summary of gastrointestinal perforation in patients with Wegener granulomatosis reported English language literature until 2023.

Reference/yr	Age/sex	ANCA	E	L	K	Perforation site	Perforation period	Pathology	Drug	Prognosis	Citation
McNabb et al (1982)	50/F	NS	+	+	−	Ileum	B	NS	P + C	Survival	^[14]^
Geraghty et al (1986)	46/M	NS	−	+	+	Ileum, colon	B	Ulceration	P + C	Death	^[15]^
Tokuda et al (1989)	46/M	c-ANCA	+	+	−	Ileum, colon	B	vasculitis	P + C	Death	^[16]^
Ushiyama et al (1997)	58/F	c-ANCA	+	+	+	Transverse colon	B	vasculitis	P + C	Survival	^[17]^
Storesund et al (1998)	28/M	c-ANCA	+	+	+	Sigmoid colon	B	vasculitis	P + C	Survival	^[18]^
Storesund et al (1998)	46/F	c-ANCA	+	+	+	Sigmoid colon	B	vasculitis	C	Survival	^[18]^
Srinivasan and Coughlan (1999)	56/F	c-ANCA	+	−	−	Ileum	B	vasculitis	P + C	Survival	^[19]^
Skaife et al (2000)	69/M	c-ANCA	−	+	+	Jejunum	A	vasculitis	P + C	Death	^[20]^
Strivens et al (2005)	54/F	PR3-ANCA	+	+	+	Ileum, colon	B	vasculitis	P + C	Survival	^[21]^
Akça et al (2005)	56/M	c-ANCA	−	+	−	Ileum	B	Ulceration	P + C	Survival	^[22]^
Shaikh et al (2006)	44/F	c-ANCA	−	−	−	Ileum, colon	B	vasculitis	P	Survival	^[23]^
Deniz et al (2007)	44/M	PR3-ANCA	+	+	−	Ileum	A	vasculitis	NS	Survival	^[24]^
Yildirim et al (2010)	32/M	PR3-ANCA	−	+	−	Ileum	B	vasculitis	P + C	Death	^[25]^
Samim et al (2010)	35/M	PR3-ANCA	+	−	+	Jejunum	B	Ulceration	P + C	Survival	^[26]^
Akbulut (2012)	47/M	NS	−	+	−	Ileum	A	NS	P + C	Death	^[13]^
Dag et al (2013)	29/M	PR3-ANCA	−	+	−	Ileum	B	vasculitis	P + C + R	Survival	^[27]^
Bulus et al (2013)	47/M	NS	−	+	−	Ileum, Jejunum	B	NS	P + C	Death	^[28]^
Dinić et al (2013)	52/F	pANCA	+	+	+	Small intestine	B	vasculitis	P	Death	^[29]^
Ruiz et al (2017)	36/M	PR3-ANCA	−	+	+	Cecum	B	No vasculitis	P + C	Death	^[30]^
Toh et al (2018)	19/F	c-ANCA, PR3-ANCA	−	+	−	Small bowel	B	vasculitis	P + R	Survival	^[31]^
Kiboshi et al (2017)	51/M	PR3-ANCA	+	+	−	Ileum	B	vasculitis	P + C + PE	Survival	^[2]^
Iwabu et al (2019)	40/M	c-ANCA	+	+	+	Colon	B	vasculitis	P + M + R	Survival	^[32]^
Sato et al (2019)	55/M	PR3-ANCA	+	−	+	descending Colon	B	vasculitis	P + C + R	Survival	^[33]^
Ulutaş (2020)	39/M	PR3-ANCA	+	+	+	Ileum	A	Inflammation	P + C	Survival	^[34]^
Levartovsky (2021)	66/M	c-ANCA	+	+	+	Ileum	B	vasculitis	P + C + R + PE	Survival	^[35]^
Our case (2023)	54/M	c-ANCA, PR3-ANCA	+	+	+	Ileum	A	vasculitis	Antibiotics	Death	_

ANCA = antineutrophil cytoplasmic antibody, C = cyclophosphamide, c-ANCA = cytoplasmic antineutrophil cytoplasmic antibody, E = ear, nose and throat leision, F = female, K = kidney involvement, L = lung involvement, M = male, M = methotrexate, NS = not stated, P = prednisolone, PE = plasma exchange, PR3-ANCA = antineutrophil cytoplasmic antibodies proteinase 3, R = rituximab.

Perforation period: A: before treatment; B: after treatment.

**Table 3:** Comparison of survival and death in patients with granulomatous polyangiitis combined with gastrointestinal perforation.

	Survival	Death	*P* value
n	17	9	
Age, median (interquartile range)	46 (37.0–55.5)	47 (41.0–53.0)	.632
Sex, male/female	10/7	8/1	.190
ANCA positive/all	16/16 (100%)	6/6 (100%)	1.000
Ear, nose, and throat lesion, n/all	13/17 (76.47%)	3/9 (33.33%)	.046
Lung involvement, n/all	13/17 (82.35%)	9/9 (100%)	.263
Kidney involvement, n/all	9/17 (52.94%)	5/9 (55.56%)	1.000
Onset of perforation, A/B	2/15	3/6	.302

ANCA = antineutrophil cytoplasmic antibody, n = number of patients.

Onset of perforation: A: before treatment, B: after treatment.

Masiak et al^[36]^ found that 9 of 34 patients with GPA had GI complications, and 1 of 5 patients with GI bleeding had an intestinal perforation. They noted that histopathological changes in the GI tract showed nonspecific inflammation that was not confirmatory of a GPA diagnosis. Pagnoux et al^[37]^ found signs of vasculitis in colon biopsies in only 3 of 62 patients with vasculitis with GI involvement, suggesting that the differential diagnosis of GI symptoms is quite difficult. Such symptoms may be related to GPA, but they may also be associated with nonspecific inflammatory diseases of the gut, such as Crohn disease. Camilleri et al^[38]^ suggested that nonspecific histopathological findings should not exclude a diagnosis of GPA because such negative findings could be due to the biopsy specimens that were collected not including deep small- and medium-sized vessels in the submucosa of the intestine.

A definitive mechanism for intestinal perforation in patients with GPA has not been clarified. It is difficult to distinguish whether perforation was caused by the underlying vasculitis itself or if the long-term use of corticosteroids and immunosuppressants may have contributed to the weakening of the intestinal wall.^[35]^ In our review of reported cases, 4 patients with GPA were treated with hormones and immunosuppressants after the occurrence of intestinal perforation.^[13,20,24,34]^ Based on his review of the literature, Akbulut found that manifestations of vasculitis were visible in 61.5% of perforation sites in histopathology. He, therefore, concluded that vasculitis plays a more important role than steroid hormones in the development of intestinal perforations in patients with GPA.^[13]^ The pathology of the ileum in our patient was suggestive of deformation, erosion, and necrosis in some areas of intestinal wall tissue and mucosa, disruption of intestinal wall continuity in focal areas, and infiltration of epithelioid and inflammatory cells, including inflammatory cell infiltration in the small vessels of the intestinal wall. These findings are consistent with ulceration in association with intestinal perforation and vasculitis changes with endothelial cell proliferation.

Excessive activation of immune cells in patients with neocoronary pneumonia has been reported to lead to elevated levels of several autoantibodies and inflammatory cytokines, including IFN-γ and TNF-α.^[39]^ Given the COVID-19 comorbidity context of the present case, it is noteworthy that cytokine activation of endothelial cells and coronavirus binding to ACE2 receptors (widely distributed in the lungs and intestines) can produce direct inflammatory responses in endothelial cells, which in turn cause vascular inflammatory changes.^[40]^ In a literature review encompassing 25 cases, Bulte et al^[41]^ found GI perforation to be a rare but dangerous extrapulmonary complication of COVID-19 with a high emergency surgery mortality.^[42]^ Local inflammatory responses caused by viral replication in GI cells may play an important role in intestinal perforation.^[43]^ There have been case reports of patients with GPA and COVID-19 receiving immunosuppressant therapy,^[44,45]^ but immunosuppressive and steroid hormone therapy treatments increase GI perforation risk and may mask the typical abdominal signs of sepsis, leading to missed diagnoses.^[41,43,44]^ It has been suggested, but not yet conclusively demonstrated, that COVID-19 may be a predisposing factor for intestinal perforation in patients with ANCA-positive GPA.^[46,47]^ The clinical course observed in our patient, who developed coronary pneumonia followed by sudden intestinal perforation a couple of months after he was admitted with GPA, is consistent with a possible COVID-19-induced vulnerability to GPA-associated intestinal perforation. In this regard, it is notable that our patient chest CT was atypical of GPA patients in that it did not reveal any ground glass changes, solid changes, or cavities.^[42]^

Good outcomes can be obtained for patients with GPA with a combination treatment of corticosteroids and cyclophosphamide. Summary of 25 cases of GPA complicated with intestinal perforation reported in the literature, 60% survival rate (9/15) for patients with GPA complicated with bowel perforation treated with corticosteroids and cyclophosphamide. One of their patients suffered an ileal perforation with diffuse peritonitis and, consequently, underwent emergency surgery to remove part of the ileum and then achieved remission following an early postoperative plasma exchange together with a combination of intravenous cyclophosphamide and glucocorticoids followed up with azathioprine maintenance therapy. Therefore, as Kiboshi et al^[2]^ emphasized that clinicians should be aware of different presentations of GPA with intestinal perforation to improve prognoses by securing an early diagnosis and appropriate intensive treatment.

Unfortunately, due to our patient refusal to accept steroid hormone and immunosuppressive therapy postoperatively, his vascular inflammatory lesions entered a progressive worsening phase and he developed splenic and renal infarcts. Notwithstanding, there are some patients who are steroid-resistant and in such patients, a standard combined therapy of steroids with cyclophosphamide may not be sufficient to achieve durable remission or prevent relapse. Biologic pharmaceuticals, such as the chimeric anti-CD20 monoclonal antibody rituximab, may potentially enable GPA patients with systemic involvement to achieve sustainable remission. Thus far, there have been only a few reports concerning the use of rituximab in GPA cases with intestinal perforation.^[27,31–33,35]^ In 1 case, a patient with GPA complicated with colonic perforation obtained postoperative relief with rituximab, without notable adverse effects, after not responding well to conventional treatment.^[33]^ Another patient with GPA who had multiple perforations of the distal ileum exhibited a good clinical outcome when given conventional treatment together with rituximab.^[27]^ Further studies, particularly randomized trials, are needed to verify the effectiveness of biologics such as rituximab for GPA with intestinal perforation.

In summary, the prognosis of GPA patients with GI perforation may be improved by early diagnosis and treatment with steroid hormones and immunosuppressants. However, currently, the paucity of reports of intestinal perforation in GPA patients infected with COVID-19 precludes a full analysis of COVID-19 interactions with GPA. The accumulation of such cases in the future can further provide clinicians with treatment guidance.

## Acknowledgments

We are grateful to Dr Cheng-mao Xia and Kang Liu. Cheng-mao Xia is a pathologist in the Department of Pathology for providing the pathological pictures. Kang Liu is a radiologist in the Department of Medical Imagingfor providing the CT images.

## Author contributions

**Data curation:** Xiaofeng Zheng.

**Formal analysis:** Huijuan Shao.

**Funding acquisition:** Jiucong Zhang.

**Investigation:** Wenbo Li, Peng Cheng.

**Methodology:** Huijuan Shao.

**Resources:** Zhen Qian, Jie Yang.

**Writing – original draft:** Huijuan Shao, Dong Liu.

**Writing – review & editing:** Huijuan Shao, Dongmei Liu, Jiucong Zhang.
